# Side-stepping the guardian of the genome: current cancer therapeutics targeting mutant p53

**DOI:** 10.3389/fphar.2025.1529483

**Published:** 2025-01-29

**Authors:** Iulianna C. Taritsa, Eric T. Fossel

**Affiliations:** VISKA Bio, Cambridge, MA, United States

**Keywords:** oncology, p53, immuno oncology, therapeutics, cancer, immunogenic cell death

## Abstract

Cancer therapies have attempted to target the transcription factor p53, a gene also described as the “guardian of the genome,” for decades. However, the approach has faced numerous barriers to clinical efficacy due to several factors: mutations in p53 occur in almost half of all human cancers, mutations are cancer-specific, and the associated genomic changes grant mutant p53 with oncogenic potential unique from that of wild-type p53. A host of new therapeutic agents have emerged that work to target mutant p53. These agents can broadly be classified into six categories: the viral approach, direct modifiers of the p53 pathway, epigenetic modifiers of the p53 pathway, synthetic lethal agents, structural reactivators, and immune activating vaccines. Even these strategies have been met with limited success. Bypassing p53 entirely may be the next avenue in cancer therapeutics to kill tumor cells regardless of p53’s mutation pattern.

## Introduction

Tumor protein p53 (TP53) has long been recognized as one of the most important genes in regulating cell death, and has been called the “cellular gatekeeper” or “the guardian of the genome” (Lane, 1992; Levine, 1997). p53 is a tumor suppresser gene and its translated protein. When the transcription factor recognizes abnormal DNA, abnormal tubulin, or other abnormalities which could result in cancer, p53 plays a key role in initiating a cascade of events which results in cell death.

P53 as its translated protein is a 393 amino acid tetramer composed of several domains. These include DNA-binding and tetramerization domains as well as highly mobile, less structured domains (Cho et al., 1994; Clore et al., 1995; Bell et al., 2002; Jeffrey et al., 1995; Veprintsev et al., 2006). The DNA-binding core domain of the protein has been extensively characterized, as it is the most frequently mutated in cancer cells. Its surface is two loops that are stabilized by zinc ion and a loop-sheet-helix motif. The amino acid residues most frequently mutated within the core DNA-binding domain are referred to as either contact or structural residues, depending on their role in maintaining the structural integrity of the surface (Olivier et al., 2002). The hotspot mutations in the p53 protein have been studied via NMR and X-ray crystallography due to their oncologic potential (Joerger et al., 2005; Wong et al., 1999). These common polymorphisms have been shown to create characteristic local structural changes (Wong et al., 1999). Most notably, oncologic polymorphisms of active study include the structural mutants G245S and R249S. Loops L2 and L3 are the most commonly structural altered regions, which are hypothesized to disrupt the DNA-binding surface (Milner, 1995).

While many aspects of the p53 pathway are still under active investigation, it is clear that the p53 protein regulates the production of a host of proteins and microRNAs that then feedback to control p53 expression (Ghosh et al., 2004). Because of its function as both an activator and inhibitor of its own pathway containing a large host of genes critical to cellular survival, the p53 network is complex. As a tumor suppressor, wildtype p53 controls cell death in a highly redundant fashion, regulating five forms of cell death: 1) apoptosis, 2) ferroptosis, 3) necroptosis mediated by TNF, 4) necroptosis mediated by FAS ligand, and 5) senescence with an associated memory immune response (Levine, 2020b). The p53 program is influenced toward certain pathways of cell-cycle arrest through intrinsic and extrinsic stressors and degrees of several epigenetic modifiers (Levine, 2022; Tomicic et al., 2021).

Wildtype (WT) p53 in a healthy cell is expressed at very low levels, to prevent premature death. Expression is controlled via a negative feedback loop between WT p53 and Mouse double minute two homolog (MDM2), also known as E3 ubiquitin-protein ligase (Barak et al., 1993). The *Mdm2* gene is a transcriptional target of the p53 protein and its product ubiquitinates p53 which renders it susceptible to degradation by proteosomes (Wade et al., 2010). Regular degradation keeps p53 expression down and maintains normal homeostasis. After cells with native p53 are exposed to extracellular or intracellular stressors, such as high levels of reactive oxygen, oncogene expression, nutrient or ribonucleotide depletion, or irradiation, the role of p53 is to help the cell detect DNA damage and inhibit further proliferation if necessary (Kruse and Gu, 2009; Mandinova and Lee, 2011; Laptenko and Prives, 2006). There is a strong association between WT p53 and microtubulin, such that microtubule dynamics regulate p53 levels and p53 signaling can influence microtubule activity and remodeling (Galmarini et al., 2003; Giannakakou et al., 2002; Giannakakou et al., 2000; Kim et al., 2013; Mabjeesh et al., 2003). Wildtype p53 in healthy cells accumulates in response to disrupted microtubule dynamics that signify increased cell stress. Alteration of microtubule function ultimately increases p53-dependent apoptosis (Giannakakou et al., 2002).

The source of cellular stress varies greatly, though in the classical model for p53 activation, the tumor suppressor gene will undergo three sequential activation steps. First, there is stress-induced stabilization of p53, typically through phosphorylation by kinases. This phosphorylation inhibits MDM2 from associating with p53. Second, the stabilized p53 protein accumulates and can now bind to its transcriptional activation site on the *p53* gene. Third, the DNA-bound p53 recruits additional transcriptional machinery to activate the transcription of target genes in the p53 pathway (Kruse and Gu, 2009). The activation of its target genes, such as p21 (a promoter of cell cycle arrest) and pro-apoptotic proteins Bax (Bcl-2-associated protein), PUMA (p53 upregulated modulator of apoptosis), and Noxa (phorbol-12-myristate-13-acetate-induced protein 1), culminate to decide the cell’s fate: senescence or regulated death (Laptenko and Prives, 2006; Raycroft et al., 1990).

Briefly, p53 can control apoptosis through a separate cascade related to reactive oxygen species. It transactivates redox-related genes and initiates an intracellular generation of reactive oxygen species (ROS) which is toxic to the cell. The high levels of ROS within the cell kickstart the degradation of mitochondrial outer membranes as well as its lysosomal membranes, which then incites the cell’s mitochondria to release more ROS and the lysosome to release cellular degradation process. The result is the death of the cell (Polyak et al., 1997).

Other forms of cell death processes, such as ferroptosis, have also been shown to be p53 dependent. Ferroptosis, which is a form of regulated cell death initiated by oxidative perturbations of the intracellular microenvironment that is under control by GPX4 and able to be inhibited by iron chelators and lipophilic antioxidants, is a complex process that has been linked to cancer, aging and degenerative disease, and malaria (Liu and Gu, 2022b; Galluzzi et al., 2018). There are several core metabolic elements in ferroptosis – including iron levels, ROS, GPX4/p21, FSP1, iNOS, GCH1, and iPLA2β, among others – that are tightly linked to tumor biology, and each has been demonstrated to be affected by p53 expression (Dixon and Stockwell, 2019; Liu and Gu, 2022a; Venkatesh et al., 2020; Soula et al., 2020; Kapralov et al., 2020; Malley et al., 2018). Ferroptosis pathways previously thought to be p53-independent, have recently shown to be dependent on transcription factors such as TFE3 and TFEB, such that increased levels of TFE3/TFEB can decrease ferroptosis (Wang et al., 2021; Chen et al., 2023). In fact, these transcription factors in separate studies have been shown to be activated in a p53-dependent manner (Brady et al., 2018). Thus, p53 remains tightly linked to ferroptosis in both its canonical and noncanonical pathways via its regulation of lipid, iron, ROS, and amino acid metabolism.

In this review, we will discuss the challenges faced by therapeutics aimed at p53, either in its wildtype or mutant/null forms. Novel strategies of targeting tumors by bypassing p53 entirely as a method to overcome the various roadblocks encountered by p53-focused therapies are discussed and improve the current understanding of the immuno-oncology therapeutic landscape.

## Wildtype p53 and cancer

While the tumor suppressor protein is often inactivated in cancer cells to allow for increased proliferation, the wildtype p53 is retained in certain cancers (Kim et al., 2009). In these cases, the pro-survival effects of p53 become the dominant driving force to cellular longevity. Specifically, p53 in these cells directly activates genes with anti-apoptotic activity and promotes maintenance of low levels of reactive oxygen species to prevent cell death (Kim et al., 2009). For example, in a subtype of glioblastoma multiforme (GBM) known as primary GBM, 70% of glioma cells express wildtype p53 and these cells have been observed to have a selective impairment of the apoptotic functions of WT p53, while still being able to regulate p53 control over DNA repair and control of cell cycle (Shu et al., 1998).

The second mechanism whereby wildtype p53 helps the cancer cell proliferate is in suppressing mitochondrial overproduction of reactive oxygen species. This mechanism has been well-demonstrated in hepatocellular carcinoma (HCC). In hepatocellular cancer cells, it was been shown that WT p53 promotes increased p53 upregulated modulator of apoptosis (PUMA) expression, consequently decreasing pyruvate uptake in the mitochondria, which then decreases cytotoxic ROS generation and helps these tumor cells survive (Kim et al., 2019).

Newer studies have shown that wildtype p53 is an important regulator of autophagy, a process by which the cell degrades and recycles its own organelles in response to stress (Shim et al., 2021). p53 can promote or inhibit autophagy depending on its subcellular localization, mutation status, and amount of stress in the cellular environment. Without stress and in physiologic conditions, wildtype p53 inhibits autophagy though when stress levels are high, p53 works oppositely to promote autophagy through activation of the mTORC1 pathway (Feng and Levine, 2010). Nuclear p53 can also promote autophagy through direct transcriptional activation of autophagy-related genes, such as DRAM1 and LCB3 (Crighton et al., 2006; Scherz-Shouval et al., 2010). P53-mediated metabolic shifts can also regulate autophagy through MDM2 (Duan et al., 2015). The regulation of autophagy has major implications for cell survival, and thus, in cancer models, the dual role of p53 may become apparent. Cancer cells with either altered metabolism, increased cellular stress, certain amplified genes in autophagy-related pathways, may have greater chance of evading p53-dependent autophagy and subsequently avoiding cell death (Lacroix et al., 2020; Liu and Gu, 2022a). In other models, autophagy can protect cancer cells from wildtype p53-mediated apoptosis (Fitzwalter et al., 2018; Shim et al., 2021). The nuanced duality of wildtype p53’s role in regulating autophagy has significant implications for cell survival.

p53 has long been a popular target for all kinds of anti-cancer therapies, because the inactivation of p53 functions is an almost universal feature of human cancer cells (Lane et al., 2010). The desire to focus on wildtype p53 was fueled by early experiments showing the ability of WT p53 restoration in previously cancerous cells to trigger cancer cell death or make these cells susceptible to chemotherapies (Ventura et al., 2007). Yet, the graveyard of wildtype p53 anti-cancer therapeutics is vast, with scientists attributing lack of success to a variety of factors, most often citing the negative crosstalk with mutant p53 where presence of surrounding mutant p53 passed genome instability and mutating ability to targeted cells which allowed them to escape effects of wildtype p53 (Vinyals et al., 1999; Zeimet and Marth, 2003).

## The problem with the traditional approach: variations in p53

The critical problem with traditional cancer treatments targeting wildtype p53 is that almost fifty to sixty percent of all cancers have at least one mutated copy of p53 making them resistant to treatments with wildtype p53-directed therapies (Kandoth et al., 2013).

Mutations in p53 are most commonly missense mutations which allow for transcription of full-length mutant proteins with a single amino acid substitution (Brosh and Rotter, 2009). This mutation type sets p53 apart from other tumor suppressor genes whose mutations are frequently deletions or nonsense and cause little to no protein expression. The vast majority (80%–90%) of p53 missense mutations are in the DNA binding domain, and more specifically at six major hotspots: R175, G245, R248, R249, R273 and R282. Estimates for other types of p53 mutation are that 5%–7% are nonsense, another 5%–7% are frame-shift, and less than 5% respectively are in-frame insertions/deletions and synonymous mutations (Leroy et al., 2014). Mutations in p53 lead to loss of wild-type p53 functions and also form hetero-tetramers with any remaining wildtype p53, producing a dominant negative effect. Late-stage tumors that start with only one mutant p53 allele often have complete loss of wildtype p53 ultimately due to loss of heterozygosity on the second allele (Song et al., 2007). Many p53 mutants also develop gain-of-function (GOF) actions and become able to interact with other transcription factors such as p63, p73, NF-Y, Sp1, ETS1/2, NF-κB, ATM, and SMADs, which are transcription factors involved in cell cycle and apoptosis. The GOF mutations allow the tumor cells to proliferate at an accelerated rate, and some mutants become more resistant to chemotherapy or radiation (Amelio and Melino, 2020; D'Orazi and Cirone, 2019; Hanel and Moll, 2012; Liu et al., 2010). Looking at the mutational burden of p53, it becomes clear that almost all cancers have some form of compromised p53-related activities. Thus, many cancers are undruggable by wildtype p53-directed therapies.

For therapies that do attempt to target mutant p53, there are several challenges. First, restoring wildtype p53 in cancer cells with loss of function (the dominant negative type of tumor) is more difficult than inhibiting gain of function mutants, like oncogenic kinases. Because the p53 pathway is inactivated by deletion of mutation of p53 or by inhibitor overexpression (e.g., Mdm2 or Mdm4 overactivation), reactivating the WT p53 must be specifically designed to the genetics of a specific tumor type, depending on the method by which p53 is inhibited. For example, for tumors where the primary mechanism of low p53 activity is through elevated inhibitor levels, pharmaceutical agents will need to target the inhibitors or the binding of the inhibitors to p53. On the other hand, for tumors with higher levels of p53-null mutant types, therapies must either reintroduce p53 or convert the null-p53 to wild type (Lozano, 2019). These therapies require detailed knowledge of the method of p53 inactivation in each tumor type of interest.

Other factors that make mutant p53 difficult to target is that every p53-mutant allele examined in the literature differs from the other documented p53 mutants. Of the over three hundred p53 mutants observed in cancers, each mutation occurs at different frequencies and contributes differently to how resistant a cancer is to other treatments (Levine, 2022). In cancers caused by viral inactivation of p53, the mode of inactivation is through protein-protein interactions rather than through native mutations burden, which makes these viral-induced cancers (e.g., cervical cancer through the HHV19 and HHV21 viruses) hard to restore WT p53 function in. Finally, several cancers are resistant to cancer therapies that target WT p53, like acute promyelocytic leukemia (PML). Current treatments for PML rely on some small proportion of cells to express WT p53 such that the arsenic trioxide plus all-trans-retinoic acid can modify the WT p53 protein from a repressor to an initiator of senescence, which is necessary to either kill the PML cells or kickstart the differentiation of PML cancer cells to neutrophils (Levine, 2022).

To add to the challenges surrounding researchers’ understanding of what drives alterations in p53, newer studies have shown that the gut microbiome may also play an important role. For example, reports have shown the microbiome can affect the functions of mutant p53 to promote intestinal tumor formation (Kadosh et al., 2020; Lau and Yu, 2020). Specifically, certain metabolites and acids such as gallic acid may affect the function of mutant p53, possibly switching its role from tumor suppressor to oncogene and *vice versa* (Kadosh et al., 2020; Wong and Yu, 2019). Ultimately, many factors influence the structure and function of p53, making it an incredibly difficult protein and gene to target.

## Novel solutions and strategies to target mutant p53

**FIGURE 1:**
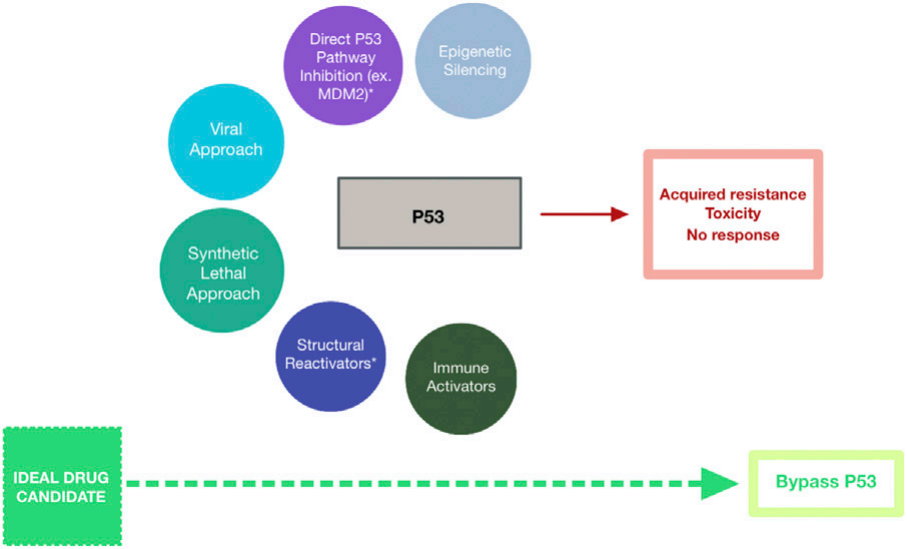
Schematic Representing Strategies Targeting Mutant p53 for Cancer Treatment. Drug candidates have included epigenetic silencers (blue-grey), direct p53 pathway inhibitors (purple), mutant p53-targeting viruses (cyan), synthetic lethal agents (teal), structural reactivators (navy), and immune activators (dark green). These strategies have been met with minimal success due to acquired resistance among cells expressing mutant p53, toxicity of the therapeutic agent against healthy cells, and overall lack of response to the agent. Bypassing p53-targeting entirely (lime green) may represent the path to successful cancer treatment.

Despite the numerous challenges of targeting mutant p53 in cancer therapies, scientists and commercial pharmaceutical programs have attempted in multiple ways to circumvent the associated hurdles with novel therapeutic agents (Figure 1). None have been especially successful but are worth noting (Table 1).

**TABLE 1:** Therapeutic agents that have been aimed at targeting p53 in its various mutant forms are described. Mechanism of action include epigenetic silencers, direct p53 pathway inhibitors, mutant p53-targeting viruses, synthetic lethal agents, structural reactivators, and immune activators. The cancer type(s) where the agent has been tested or has entered clinical trials are also listed.

Therapeutic	Mechanism of action	Cancer type(s)
ONYX-015 (Onyx Pharmaceuticals)	Viral Approach (adenovirus)	Head/neck squamous cell carcinoma, malignant gliomas, pancreatic, ovarian, colorectal, metastatic lung
Gendicine (Shenzhen SiBionoGene Technology rAD-p53)	Viral Approach (adenovirus)	Head/neck squamous cell carcinoma
Vogelstein et al	Viral Approach (adenovirus)	Colorectal
CF33-hNIS VAXINIA (City of Hope)	Viral Approach (vaccinia virus)	Colorectal, lung, breast, ovarian and pancreatic
RG7112 and RG7118 (Roche)	Direct p53 Pathway Inhibition (MDM2)	Acute myeloid leukemia, chronic lymphocytic leukemia, small cell lymphocytic leukemia
SAR-405838 (Sanofi)	Direct p53 Pathway Inhibition (MDM2)	Osteosarcoma, acute leukemia, prostate, colorectal
APG-115 (Ascentage)	Direct p53 Pathway Inhibition (MDM2)	Melanoma, peripheral nerve sheath tumor, liposarcoma, non-small cell lung cancer *ATM* mutation solid tumors, urothelial
AMG-232 (Brown University)	Direct p53 Pathway Inhibition (MDM2)	Multiple myeloma, liposarcoma, glioblastoma multiforme, breast cancer
NVP-CGM097 (Novartis)	Direct p53 Pathway Inhibition (MDM2)	Squamous cell carcinoma, non-small cell lung cancer (NSCLC), acute myeloid leukemia, neuroendocrine tumors
HDM201 (Novartis)	Direct p53 Pathway Inhibition (MDM2)	Acute leukemia, wt-p53 solid tumors
RG7388 (Roche)	Direct p53 Pathway Inhibition (MDM2)	Acute myeloid leukemia
Azacytidine and Decitabine	Epigenetic Silencer	Acute myeloid leukemia, myelodysplastic syndromes
Arsenic Trioxide (NCT04869475, NCT04489706, NCT04695223)	Epigenetic Silencer	Promyelocytic leukemia, endometrial, ovarian
400945 (Treadwell Therapeutics)	Synthetic Lethal Agent	Acute Myeloid Leukemia, Myelodysplastic Syndromes, chronic myelomonocytic leukemia
AZD1775 (Merck)	Synthetic Lethal Agent (Wee-1)	NSCLC, Ovarian
ZNL-02-096 and ZN-c3 (Zentalis)	Synthetic Lethal Agent (Wee-1)	Osteosarcoma, gynecologic tumors
CP-31398 (Pfizer)	Structural Reactivator	Glioma
CDB3 (Friedler et al)	Structural Reactivator	N/A (studied for p53 rescue applications)
APR-246 (Aprea Therapeutics)	Structural Reactivator	Acute myeloid leukemia, Myelodysplastic Syndromes
COTI-2 (MD Anderson)	Structural Reactivator	Triple-Negative Breast Cancer, Head/neck squamous cell carcinoma
MVA-based Vaccines (NCT03113487; NCT02432963)	Immune Activator	Ovarian, Peritoneal, Fallopian Tube; NSCLC, head/neck squamous cell, hepatocellular, renal cell, melanoma, bladder, soft tissue sarcoma, triple-negative breast, pancreatic, colorectal
HLA-A2 DC Vaccine (Barfoed et al)	Immune Activator (DC Vaccine)	SCLC
Indoximod (Solimon et al)	Immune Activator (DC Vaccine and adenovirus)	Breast, Colon, Gastric, Lung, Tongue, Ovarian, Chondrosarcoma

### The viral approach

There have been a handful of trials which use viruses to target missense p53. One technique is the use of mutant viruses that replicate only in cells that contain mutant p53, and consequently aim to kill only those cells. ONYX-015 was the first oncolytic therapy to reach human clinical trials. It consisted of a human adenovirus genetically engineered to incorporate deletions in the E1B-55k and E3B regions, which suppress p53 (Opyrchal et al., 2009). However, in clinical trials of ten patients with solid tumors metastatic to the lung, only one patient was found to have intratumoral virus detected. In a clinical trial of 18 patients with refractory colorectal cancer, 36% of patients were found to be positive for virally infected cells but among those patients, one died of disease progression with the viral agent seen in the spleen and normal liver on autopsy, with very low levels of virus seen in the tumor itself (Kaufman and Bommareddy, 2019). This raised questions on how effective the viral agent was at infecting cancer cells alone. Additionally, no antitumor responses were seen even after intratumoral administration in patients with pancreatic cancer or after intraperitoneal administration in patients with ovarian cancer (Vasey et al., 2002; Hecht et al., 2003). Further trials of ONYX-015 were not pursued.

Shenzhen SiBionoGene Technology similarly created a recombinant human adenovirus with a WT p53 tumor suppressor gene. Their studies show that the viral vector can infect human head and neck tumor cells and induce normal p53 expression, which effectively suppresses tumor cell growth. However, the effect of these therapies to kill tumor cells is unclear. Though their studies show that, when combined with traditional chemotherapy regimens, patients treated with rAD-p53 live longer post-diagnosis, the interpretation of these results must be put in the context of the finding that p53 mutation status did not significantly influence efficacy outcomes and long-term survival rate for Ad-p53-treated patients (Zhang et al., 2018). Therefore, it is unclear how specific these vectors are at targeting mutated p53.

The research team of Dr. Burt Vogelstein also tried to use adenovirus vectors to induce cell death in tumors expressing mutant p53. They worked to induce apoptosis in the colorectal cancer cell line DLD-1, which contains an inactive WT *p53* gene (Ad-p53). The group infected these cancer cells with an adenovirus that halted replication in infected cells. Experiments showed mixed results, with limited cell killing (Polyak et al., 1997).

Another technique being pursued is to immunize patients with a viral vector which trains the immune system to act against p53 epitopes. At City of Hope, a clinical trial was started to immunize patients who have ovarian cancer with a vaccinia virus vector against p53 epitopes, e.g., p53 MVA. No results have been published from this clinical trial yet, though it has been theorized that the B-cell adaptive immune response triggered by this approach may not be sufficient to inhibit tumor growth (Levine, 2020b; Levine, 2020a).

### Modification of the p53 pathway: Directly or through epigenetics

Modification of the p53 pathway, either directly or through epigenetics, is another method researchers have used to try to target cancer. To target the p53 pathway directly, various approaches have been tried. Many center around MDM2, which regulates the expression of p53 protein through negative feedback. Specifically, the protein binds to the p53 protein which weakens its transcriptional function and has a unique RING domain which helps p53 move out of the nucleus and into the cytoplasm thus decreasing p53 transcription function (Bang et al., 2019; Hock and Vousden, 2014). The inhibition of p53-MDM2 complexes is one direct manipulation of the p53 pathway where the blocking of this protein-protein interaction by small molecules or stapled peptides stops the negative feedback cycle and increases WT p53 levels. Such increased activity of WT p53 has been shown to cause remission of some tumors in several tissue types.

To date, there have been several MDM2 inhibitors with different structural types that have gone through clinical trials. These include RG7112 and RG7118 from Roche, SAR-405838 and APG-115, AMG-232, NVP-CGM097, and HDM201. These small molecules have reached Phase I and II trials for chronic myeloid leukemia (CML), acute myeloid leukemia (AML), solid tumors, and hematological tumors (Zhu et al., 2022). Recently, the MDM2 inhibitor RG7388 was developed and hit Phase III clinical trials for refractory AML, the first of the MDM2 inhibitors to reach this milestone (Montesinos et al., 2020). However, as a class, the MDM2 inhibitors have been met with several barriers to success. The reason is because these drugs work only with fractions of cancers that have WT p53 and not with those with mutant p53 proteins. Also, these drugs work on normal cells, including hematopoietic stem cells and rapidly re-populating gastrointestinal cells, thus there are many adverse on-target side effects that have limited use. The MDM2 inhibitors also lead to upregulation of non-MDM2 ubiquitin ligases, which degrade p53 and ultimately lead to acquired resistance. These mutations in p53 that lead to resistance to MDM2 inhibitors also has the potential to promote progression of any tumor present (Aziz et al., 2011; Hoffman-Luca et al., 2015; Jones et al., 2012). Other inhibitors of p53 pathway interactions, with associated proteins in the pathway including MDM4 and PPMID-1, have relied on similar principles of trying to raise WT p53 in cells in order to decrease dysfunctional p53 transcription driving tumor burden (Esfandiari et al., 2016; Pishas et al., 2015). These therapies have been met with poor results for the same reasons as the p53-MDM2 complex inhibitors – the reliance on WT p53 and the toxic side effect profile make these candidates either ineffective or not safe.

Another route pursued to decrease dysfunctional p53 in tumors is through epigenetic modification of the p53 pathway. There is clinical evidence that chemotherapeutic agents azacytidine or decitabine can work to change CpG methylation in the genome or in ribosomal RNA specifically for p53 or related proteins expression. Experiments show the epigenetic modifications induced by these two drugs cause superior inhibition of cell proliferation in cells with p53 mutations than in cells with WT p53 (Levine, 2020a; Levine, 2020b). In NCT03855371, a phase I trial with five patients with AML or myelodysplastic syndrome who were treated with decitabine and arsenic trioxide, there was remission in tumor burden (NCT03855371). A trial of patients with AML who harbored p53 mutations treated with decitabine alone had similar results (Welch et al., 2016). Epigenetic silencers of cells with mutated p53 have been attempted to treat refractory solid tumors, recurrent and metastatic ovarian and endometrial cancer (NCT04869475, NCT04489706, NCT04695223). Unfortunately, these trials ultimately had patients relapse and develop resistance to these agents. It is hypothesized that epigenetic-centered therapies have failed because the cancers rapidly mutate to escape the methylation-induced silencing. The findings support the concept that wildtype p53 is protective against significant epigenetic changes.

### Synthetic lethal agents

In cancer therapeutics, synthetic lethality targets cells with high mutational burden. The paradigm is based on the idea where the inhibition of two genes is cytotoxic to the cell, while the inhibition of either gene individually is not. In cancers where there are inactive genes, therapies can target the synthetic lethal partner gene to exert its effect (Zhang et al., 2021). This is useful in cancers where the mutation causes loss-of-function. The synthetic lethal principle has been successful in treating ovarian and breast cancers harboring well-known mutations BRCA and PARP (Tewari et al., 2015).

To target mutant p53, there are several agents in trials acting on the synthetic legal principle, with limited success. For example, Treadwell Therapeutics developed an agent known as 400,945 to target acute myeloid leukemia cells with mutant p53 and high levels of aneuploidy. Drug 400,945 inhibits PLK-4, an enzyme important in the G2-M checkpoint step of cell division by sensing damaged chromosomal DNA (Veitch et al., 2019). In cells with mutant p53, inhibited PLK-4 combined with higher numbers of centromeres seen in mutant p53 cells causes drug-treated cells to develop aneuploidy and die quickly after (Jonas et al., 2022). Preliminary Phase 2 results in patients with acute myeloid leukemia, chronic myelomonocytic leukemia, and myelodysplastic syndrome with mutated p53 show no responses of cancer to therapy to date (Jonas et al., 2022).

Other G2-M checkpoint inhibitors, specifically targeting the wee-1 protein kinase, continue to be pursued to kill mutant p53 cancers. Wee-1 inhibitor AZD1775 was shown to cause cell death in KRAS-mutant non-small cell lung cancer. The efficacy of tumor NSCLC elimination in a mouse xenograft model was demonstrated. In these studies, AZD1775 inhibited the G2 checkpoint arrest and sensitized p53-mutant cells to DNA-damaging agents (Ku et al., 2017). The drug enhanced carboplatin efficacy in p53-mutant ovarian cancer as well (Leijen et al., 2016). At the time of this article, two other Wee1 inhibitors are also being tested: ZNL-02-096 and ZN-c3 (Tolcher et al., 2021; Topatana et al., 2020). Together, these agents represent the potential ability for synthetic lethal agents to cause selective killing in mutant p53 cells.

### Structural reactivating agents

To target tumors that have lost p53 tumor suppressor activity, some groups have isolated or created small molecules able to manipulate mutated p53 protein back to its wildtype function. Several synthetic peptides have been created that use this concept to restore mutant p53 to a nontumorigenic form. The first discovered p53 reactivator is CP-31398. This small molecule was developed in 2003 and works by binding to several mutated sites on the p53 protein, specifically V173A, S241F, R249S, and R273H. These sites are all DNA-binding domains, and once bound by CP-31398, the p53 protein is able to enter its natural conformation (Wischhusen et al., 2003). Original studies were used to kill glioma cancer cell lines. However, early work illustrated pitfalls in the approach. Cell death induced by the CP-31398 structural reactivator worked through two pathways: an early pathway that was p53-dependent and required new p53 protein synthesis, and a late p53-independent pathway that killed cells via free radical formation. Ultimately, the CP-31398 small molecule reactivator was inconsistent and further refinement was needed to isolate only p53-independent cell death (Wischhusen et al., 2003).

Another example of a structural reactivator of p53 is CDB3. Developed by Friedler et al., CDB3 is a synthetic peptide derived from 53BP2, a p53 binding protein (Friedler et al., 2002). The proposed mechanism of action is that the compound rescues conformationally compromised mutant forms of p53 by binding only to naïve, not denatured, p53 protein. After binding to the core domain of p53, CDB3 stabilizes the tumor suppressor (Nikolova et al., 2000). This small molecule was shown to successfully restore DNA binding activity to a highly unstable p53 mutant I195T back to its wild-type function. However, this therapy has faced challenges in study trials due to competitive antagonism from gadd45 DNA (Friedler et al., 2002).

Structural reactivators have become especially popular in recent years, leading to a wide array of candidate molecules (Boeckler et al., 2008; Bykov and Wiman, 2014; Demma et al., 2010; Bykov et al., 2002). Some of these have entered clinical trials. APR-246 (eprenetapopt) is an especially promising candidate which is now combined with azacytidine to treat myelodysplastic syndromes in select patients. Clinical trials (NCT 03072043) have showed higher rates of complete remission in these patients (Sallman et al., 2018). COTI-2 is the only other p53 reactivator, at the time of this review, to have entered clinical trials (Synnott et al., 2020). The drugs that have come into clinical trials have faced similar problems as the original p53 structural reactivators, with effects from the small molecules that include both p53-dependent as well as p53-independent toxicities (Peng et al., 2013). Structural reactivators need to become more targeted before they can become reliable therapeutic options.

### Immune activating p53 vaccines

Selective killing of p53-mutant cells by activating the immune system against certain variants has been attempted. In clinical trial NCT03113487, a modified vaccinia virus Ankara (MVA) vaccine was designed for patients with recurrent ovarian, primary peritoneal, or fallopian tube cancer (City of Hope Medical Center, 2023). A very similar candidate also using MVA was tested in NCT02432963, but against non-small cell lung, head and neck squamous cell, hepatocellular, renal cell, melanoma, bladder, soft tissue sarcoma, triple-negative breast, pancreatic and colorectal cancer (Chung et al., 2018). Both vaccinia-virus based therapies code for WT p53 and theoretically work to raise cellular immunity against cancer cells that contain highly excessive amounts of p53. The MVA therapeutic candidates are intended to generate antigen expression and present antigenic determinants on different HLA molecules (Hardwick et al., 2018). Once immunized with the full-length wild type p53 antigen, patients injected with the viral vector were studied for CD8^+^ and CD4^+^ T cell response. Both clinical trials also co-administered pembrolizumab. Early results have been mixed with only two patients showing p53-specific CD8^+^ T cell responses, while four patients had rapidly progressive disease (Chung et al., 2018).

Modified autologous dendritic cells represent another immune-activating vaccine approach. This method uses dendritic cells infected with viral vectors expressing p53 peptides controlled with viral promoters (Soliman et al., 2018). Abnormal accumulation of dysfunctional p53 protein in cancer cells has been attempted to be utilized for a therapeutic advantage with p53 DC vaccines to train T cells to recognize and activate against the excess p53 epitopes displaced on MHC Class I/II. For example, p53 DC vaccines were used to target the squamous carcinoma cell line SCC9 and the breast cancer cell line MDA-MB-468 (Barfoed et al., 2000). The DC vaccines originally showed promise in early trials, triggering p53 immune response in 57.1% of patients with SCLC after three immunizations (Antonia et al., 2006). Unfortunately, in phase II trials, there was no difference in DC-vaccine-treated patients *versus* control in response rate in SCLC (Chiappori et al., 2019). Another group tried to use indoximod, a small molecular inhibitor known to prevent T-cell anergy in tumor-draining lymph nodes, as a potentiator to the immune-activating effect (Soliman et al., 2018). Objective clinical data showed a minority of patients treated in the Phase 1/2 clinical trial had an immune response against p53 and best response was stable disease in four patients out of 39 (Soliman et al., 2018).

Ultimately, the immune-activating approach that trains the body to recognize pathogenic levels of p53 may be limited by reliance on p53 WT vs. mutational burden. It has not been shown to be a reliable therapeutic option.

### Areas for progress

To date, numerous therapeutic strategies have emerged that target cancer via p53, first by attempting to target wildtype p53 to the various techniques to block, silence, or convert its mutated forms to create a nonpathogenic phenotype. The result has ultimately been the same: cancers have evaded these agents either partially or entirely. At the time of this piece, only three therapies targeting mutant p53 have reached phase III clinical trials: the structural reactivators APR-256 and COTI-2 to treat mutant p53 myelodysplastic syndrome, and the MDM2 inhibitor RG7388 or idasanutlin for refractory acute myeloid leukemia (Montesinos et al., 2020; Sallman et al., 2018). Resistance of tumors to these agents has already been seen, for example, with RG7388 and glioblastoma *in vitro* (Berberich et al., 2019). The patient groups for which these agents are indicated are also small, as they have only been taken to phase III trials for patients with specific mutant p53 status (Sallman et al., 2018).

Researchers in the field targeting WT and mutant p53 have begun to consider developing p53-targeting therapeutic agents that are highly individualized and specific to each patient (Hu et al., 2021). This is an effort to adapt to the high amount of variation in mutational status and mutational burden in each case of p53 tumor load. The challenge remains in an individualized approach, however, that oncogenic missense p53 mutants crosstalk within the cancer pathogenesis pathways and thus, even an personalized approach targeting just one individual pathway may not be highly effective (Mantovani et al., 2019). Another suggested future strategy has been to simultaneously target p53 missense mutants together with their core downstream pathways by treating patients with combination therapy using the various mutant p53 inhibitors (Bykov et al., 2002; Bykov and Wiman, 2014; Girardini et al., 2014; Lehmann et al., 2012). By combining various strategies of mutant p53-targeting agents, there may be a higher likelihood that tumor cells may not be able to evade each agent in the cocktail. For example, by combining a structural reactivator with a mutant p53 DC vaccine, if there was a mutation that allowed the cancer cell to evade the structural reactivator, then the DC vaccine may serve as a second line of defense. Alternatively, there may be small molecule agents able to be used in combination with the mutant p53 inhibitors that can stabilize mutant p53, such as Histone deacetylase inhibitors (HDACi) or ROS scavengers (Suh et al., 2011; New et al., 2012; Girardini et al., 2014). Pre-treatment with these compounds stabilize mutant p53 and potentially allow the protein to be more reliably targeted by the other agents described. A national p53 initiative has also been suggested to direct increased resources to finding a successful mutant or WT p53 approach (El-Deiry, 2023). The areas for progress remain large, including agents to effectively kill solid tumors and a robust strategy to kill cancer cells of any p53 status, with less likelihood of acquired resistance.

## A new paradigm: bypassing p53 dependence

**FIGURE 2:**
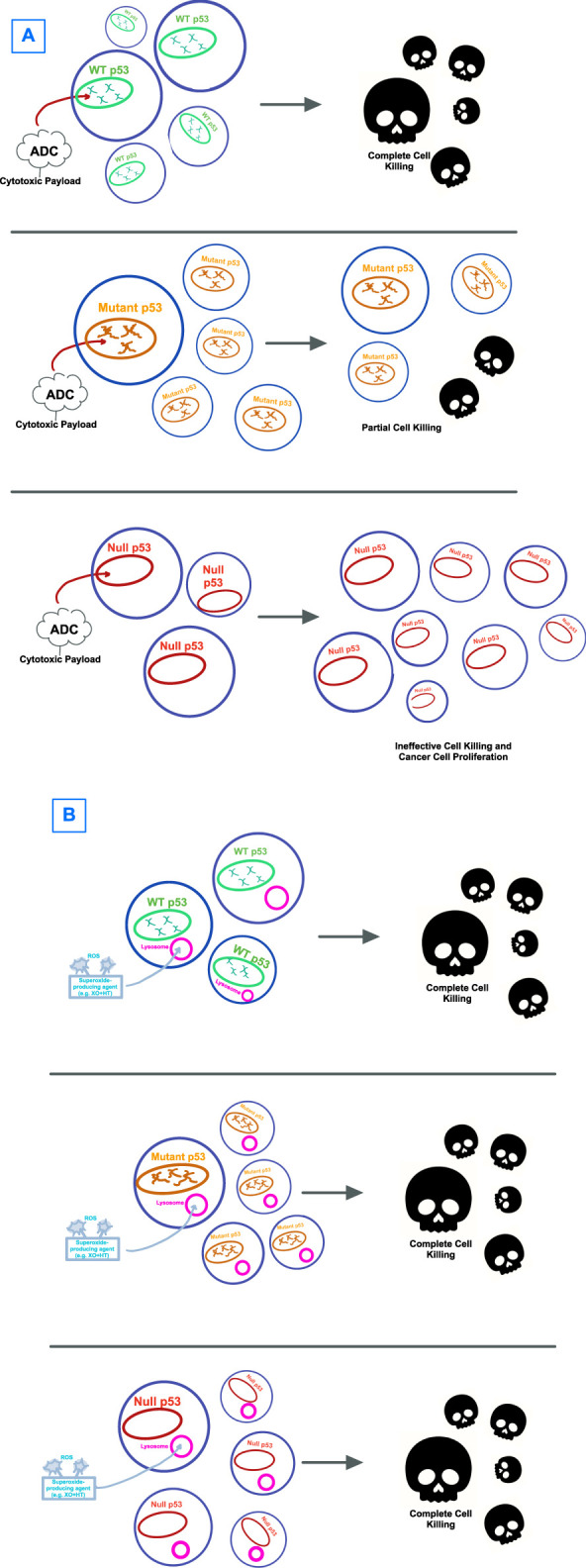
Visual hypothesis describing the failure of **(A)** p53-directed therapies *versus* **(B)** therapies that circumvent p53. **(A)** Antibody-drug-conjugates (ADCs) with cytotoxic payloads have been shown to have effectiveness against WT p53-expressing tumors, though with only limited effectiveness against mutant p53 and no to little effect on cells with null p53 mutations. **(B)** Strategies that target cancer cells by bypassing p53, with one example being through lysosome-induced immunogenic cell death shown here, have shown effectiveness in cell killing for tumor cells regardless of p53 expression.

It is possible that the dependence on p53, in any of its wildtype or mutational states, as part of a therapeutic target pathway, sets up for failure due to the heterogenous properties and high mutational burden of this protein sequence (Figure 2A). Therapeutic strategies that are p53-independent, that is, able to kill tumor cells regardless of p53’s mutation pattern, are a promising alternative. Identifying agents that are capable of cell killing without reliance on p53 gives an opportunity to determine whether p53-independent apoptosis is a common phenomenon induced by a wide host of drugs, or rather whether there are unifying, shared p53-independent pathways to apoptosis (Figure 2B).

In large screenings of drug compounds from the National Cancer Institute mechanistic sets, encompassing agents with diverse mechanisms of action that were effective in killing p53-null cells, a common theme among the agents with nonnuclear targets was the induction of lysosomal membrane permeabilization (LMP) (Erdal et al., 2005). This observation opens the question of whether LMP may be a pathway to cell death that is p53-independent. Other studies have also showed LMP and mitochondrial membrane permeabilization both working to cause cell death independent of the transcription of p53 target genes (Boya et al., 2003). It is likely that LMP may have a role in both p53-dependent and independent apoptosis due to studies showing that early LMP occurs in p53-induced apoptosis. The idea that there may be an avenue of p53-independent cell death through a lysosomal pathway is intriguing (Yuan et al., 2002).

There are certain types of cell death inducers, deemed “Type II Immunogenic Cell Death” inducers whose primary effect is not on the cell’s nucleus but on the endoplasmic reticulum directly, causing ER stress that triggers massive LMP (Krysko et al., 2012; Terenzi et al., 2016; Wang et al., 2018; Xie et al., 2022). These inducers are able to cause LMP without dependence on p53 status. One way in which these agents can work on the ER is through ROS generation, which is a well-cited cause of ER stress (Lee et al., 2022; Ma et al., 2016; Taura et al., 2013). For example, in cervical cancer cells, thioridazine (TDZ) was shown to cause Bax-Bak dependent and independent apoptosis by enhancing ROS production followed by ER stress (Seervi et al., 2018). ROS has been shown to drive ER stress and subsequent apoptosis in platinum-treated gastric carcinoma cells (Wang et al., 2017). Thus, one theorized pathway for p53 independent apoptosis is through ROS generation leading to ER stress and subsequent LMP-induced cell death (Iulianna et al., 2022).

Two documented examples of pathways involving LMP and bypassing dependence on p53 include hypericin-based photodynamic therapy (PDT) and heat shock protein (HSP) antagonists. The former approach has been tested in a variety of cancer models whereby the photosensitizer hypericin is combined with directed light treatment in red or near-infrared regions and molecular oxygen to elicit cancer cell death (Aniogo et al., 2021; Zhang et al., 2020). The mechanism relies on exogenous ROS generation created by the PDT targeted towards the tumor microenvironment, which then gets internalized and causes ER stress (Kong et al., 2020; Zhang et al., 2020). Oxidative stress to the ER and lysosome oxidative stress from photooxidation can cause lysosomal release of large numbers of proteases that help activate endogenous apoptosis-related proteins (Dai et al., 2020; Turubanova et al., 2019). The cell killing has been shown to be effective, regardless of p53 status (Lee et al., 2006).

HSP70 is a protein important for lysosomal stabilization, and thus the HSP70 antagonists have been shown to cause lysosomal leakage and p53-independent cell death. The agent apoptozole (Az), for example, has been shown to primarily translocate into lysosomes of cancer cells where Az then induces LMP and subsequent lysosome-mediated apoptosis (Park et al., 2018). Lysosomal membrane permeabilization induced by HSP70 have been effective against various osteosarcoma, breast, and pancreatic carcinoma cell lines independent of the p53-state of the tumor cells (Leu et al., 2009). The proposed mechanism of action is via the formation of p62-oligomers that can induce dysfunctional autophagy without dependence on caspase activation (Nitzsche et al., 2023).

Ultimately, the lysosome and its membrane permeabilization may play a central role in developing therapeutics that bypass the need for dependence on p53. While current drug development is focusing on antibody drug-conjugates with cytotoxic payloads like doxorubicin or antimitotic agents like maytansinol, these may prove to be ineffective because their toxic payloads do not work in p53-null or mutant cancers (Edwards et al., 2011; Sun et al., 2016). The reason is likely related to doxorubicin’s dependence on p53 in order to elicit a cancer-cell-killing effect (Wang et al., 2004). Anthracyclines’ primary mechanism of action is the intercalation into DNA, which interferes with DNA replication and activates the p53-dependent cellular stress signals (Garg et al., 2012). Thus, in cells where p53 is mutated, these drugs will be ineffective at causing the appropriate cytotoxicity of tumor cells and anticancer immune response. The localization of p53 also affects the type of cell death induced by doxorubicin: internal localization of p53 in the nucleus has been shown to leads to autophagy after doxorubicin treatment, while external localization leads to apoptosis (Mrakovcic and Fröhlich, 2018). When anthracyclines do not reach the nucleus because of defective subcellular localization, this can also effect its ability to cause cell death (Li et al., 2010). Future research is needed to develop anticancer agents whose mechanism of action is known to work without reliance on p53.

By sidestepping the guardian of the genome, we can come closer to a cancer treatment modality that is reliable and clinically effective. In an example of sidestepping p53 when it is defective, it has been shown that while doxorubicin is capable of initiating cell death through inducing lysosomal leakage, the same treatment is unable to produce lysosomal leakage and cell death in breast cancer p53-mutant cell lines (Nguyen et al., 2022). If p53 is nonfunctional a recent report shows that generation of extracellular superoxide results in uptake of oxidation products including oxidized LDL through endocytosis. This induces lysosomal leakage and immunogenic cell death (Taritsa et al., 2022). While further study is required, this finding may provide effective therapy for cancers that lack active p53 (Figure 2B).

## Conclusion

We summarize here the multitude of strategies that have been attempted to kill tumor cells via targeting of p53, either in its wildtype or mutated forms, and suggest that the reason these may have faced challenges is due to their underlying mechanism of action and target. Focusing attention on alternate routes, such as bypassing the transcription factor entirely, may result in superior clinical outcomes. P53-independent strategies, such as inducing lysosome-induced immunogenic cell death, may become an important step in dethroning the “Emperor of all Maladies”.

## References

[B1] Amelio I. Melino G. (2020). Context is everything: extrinsic signalling and gain-of-function p53 mutants. Cell Death Discov. 6, 16. 10.1038/s41420-020-0251-x 32218993 PMC7090043

[B2] Aniogo E. C. George B. P. Abrahamse H. (2021). Molecular effectors of photodynamic therapy-mediated resistance to cancer cells. Int. J. Mol. Sci. 22, 13182. 10.3390/ijms222413182 34947979 PMC8704319

[B3] Antonia S. J. Mirza N. Fricke I. Chiappori A. Thompson P. Williams N. (2006). Combination of p53 cancer vaccine with chemotherapy in patients with extensive stage small cell lung cancer. Clin. Cancer Res. 12, 878–887. 10.1158/1078-0432.CCR-05-2013 16467102

[B4] Aziz M. H. Shen H. Maki C. G. (2011). Acquisition of p53 mutations in response to the non-genotoxic p53 activator Nutlin-3. Oncogene 30, 4678–4686. 10.1038/onc.2011.185 21643018 PMC3347888

[B5] Bang S. Kaur S. Kurokawa M. (2019). Regulation of the p53 family proteins by the ubiquitin proteasomal pathway. Int. J. Mol. Sci. 21, 261. 10.3390/ijms21010261 31905981 PMC6981958

[B6] Barak Y. Juven T. Haffner R. Oren M. (1993). mdm2 expression is induced by wild type p53 activity. Embo J. 12, 461–468. 10.1002/j.1460-2075.1993.tb05678.x 8440237 PMC413229

[B7] Barfoed A. M. Petersen T. R. Kirkin A. F. Thor Straten P. Claesson M. H. Zeuthen J. (2000). Cytotoxic T-lymphocyte clones, established by stimulation with the HLA-A2 binding p5365-73 wild type peptide loaded on dendritic cells *in vitro*, specifically recognize and lyse HLA-A2 tumour cells overexpressing the p53 protein. Scand. J. Immunol. 51, 128–133. 10.1046/j.1365-3083.2000.00668.x 10652158

[B8] Bell S. Klein C. MüLLER L. Hansen S. Buchner J. (2002). p53 contains large unstructured regions in its native state. J. Mol. Biol. 322, 917–927. 10.1016/s0022-2836(02)00848-3 12367518

[B9] Berberich A. Kessler T. Thomé C. M. Pusch S. Hielscher T. Sahm F. (2019). Targeting resistance against the MDM2 inhibitor RG7388 in glioblastoma cells by the MEK inhibitor trametinib. Clin. Cancer Res. 25, 253–265. 10.1158/1078-0432.CCR-18-1580 30274984

[B10] Boeckler F. M. Joerger A. C. Jaggi G. Rutherford T. J. Veprintsev D. B. Fersht A. R. (2008). Targeted rescue of a destabilized mutant of p53 by an *in silico* screened drug. Proc. Natl. Acad. Sci. U. S. A. 105, 10360–10365. 10.1073/pnas.0805326105 18650397 PMC2492497

[B11] Boya P. Andreau K. Poncet D. Zamzami N. Perfettini J. L. Metivier D. (2003). Lysosomal membrane permeabilization induces cell death in a mitochondrion-dependent fashion. J. Exp. Med. 197, 1323–1334. 10.1084/jem.20021952 12756268 PMC2193790

[B12] Brady O. A. Jeong E. Martina J. A. Pirooznia M. Tunc I. Puertollano R. (2018). The transcription factors TFE3 and TFEB amplify p53 dependent transcriptional programs in response to DNA damage. Elife 7, e40856. 10.7554/eLife.40856 30520728 PMC6292694

[B13] Brosh R. Rotter V. (2009). When mutants gain new powers: news from the mutant p53 field. Nat. Rev. Cancer 9, 701–713. 10.1038/nrc2693 19693097

[B14] Bykov V. J. Issaeva N. Shilov A. Hultcrantz M. Pugacheva E. Chumakov P. (2002). Restoration of the tumor suppressor function to mutant p53 by a low-molecular-weight compound. Nat. Med. 8, 282–288. 10.1038/nm0302-282 11875500

[B15] Bykov V. J. Wiman K. G. (2014). Mutant p53 reactivation by small molecules makes its way to the clinic. FEBS Lett. 588, 2622–2627. 10.1016/j.febslet.2014.04.017 24768524

[B16] Chen L. Ma Y. Ma X. Liu L. Jv X. Li A. (2023). TFEB regulates cellular labile iron and prevents ferroptosis in a TfR1-dependent manner. Free Radic. Biol. Med. 208, 445–457. 10.1016/j.freeradbiomed.2023.09.004 37683766

[B17] Chiappori A. A. Williams C. C. Gray J. E. Tanvetyanon T. Haura E. B. Creelan B. C. (2019). Randomized-controlled phase II trial of salvage chemotherapy after immunization with a TP53-transfected dendritic cell-based vaccine (Ad.p53-DC) in patients with recurrent small cell lung cancer. Cancer Immunol. Immunother. 68, 517–527. 10.1007/s00262-018-2287-9 30591959 PMC6426813

[B18] Cho Y. Gorina S. Jeffrey P. D. Pavletich N. P. (1994). Crystal structure of a p53 tumor suppressor-DNA complex: understanding tumorigenic mutations. Science 265, 346–355. 10.1126/science.8023157 8023157

[B19] Chung V. M. Kos F. Hardwick N. Yuan Y. Chao J. Li M. (2018). A phase 1 study of p53MVA vaccine in combination with pembrolizumab. J. Clin. Oncol. 36, 206. 10.1200/jco.2018.36.5_suppl.206

[B20] City of Hope Medical Center (2023). P53MVA and Pembrolizumab in treating patients with recurrent ovarian, primary peritoneal, or fallopian tube cancer. Available at: https://clinicaltrials.gov/study/NCT03113487.

[B21] Clore G. M. Ernst J. Clubb R. Omichinski J. G. Kennedy W. M. Sakaguchi K. (1995). Refined solution structure of the oligomerization domain of the tumour suppressor p53. Nat. Struct. Biol. 2, 321–333. 10.1038/nsb0495-321 7796267

[B22] Crighton D. Wilkinson S. O'Prey J. Syed N. Smith P. Harrison P. R. (2006). DRAM, a p53-induced modulator of autophagy, is critical for apoptosis. Cell 126, 121–134. 10.1016/j.cell.2006.05.034 16839881

[B23] Dai Y. He F. Ji H. Zhao X. Misal S. Qi Z. (2020). Dual-functional NIR AIEgens for high-fidelity imaging of lysosomes in cells and photodynamic therapy. ACS Sens. 5, 225–233. 10.1021/acssensors.9b02090 31854187

[B24] Demma M. Maxwell E. Ramos R. Liang L. Li C. Hesk D. (2010). SCH529074, a small molecule activator of mutant p53, which binds p53 DNA binding domain (DBD), restores growth-suppressive function to mutant p53 and interrupts HDM2-mediated ubiquitination of wild type p53. J. Biol. Chem. 285, 10198–10212. 10.1074/jbc.M109.083469 20124408 PMC2856225

[B25] Dixon S. J. Stockwell B. R. (2019). The hallmarks of ferroptosis. Annu. Rev. Cancer Biol. 3, 35–54. 10.1146/annurev-cancerbio-030518-055844 PMC12851585 41613499

[B26] D'Orazi G. Cirone M. (2019). Mutant p53 and cellular stress pathways: a criminal alliance that promotes cancer progression. Cancers (Basel) 11, 614. 10.3390/cancers11050614 31052524 PMC6563084

[B27] Duan L. Perez R. E. Davaadelger B. Dedkova E. N. Blatter L. A. Maki C. G. (2015). p53-regulated autophagy is controlled by glycolysis and determines cell fate. Oncotarget 6, 23135–23156. 10.18632/oncotarget.5218 26337205 PMC4695109

[B28] Edwards A. Gladstone M. Yoon P. Raben D. Frederick B. Su T. T. (2011). Combinatorial effect of maytansinol and radiation in Drosophila and human cancer cells. Dis. Model Mech. 4, 496–503. 10.1242/dmm.006486 21504911 PMC3124055

[B29] EL-Deiry W. S. (2023). Targeting mutated p53: naivete and enthusiasm to attempt the impossible. Cancer Res. 83, 979–982. 10.1158/0008-5472.CAN-22-0995 37014041 PMC10071817

[B30] Erdal H. Berndtsson M. Castro J. Brunk U. Shoshan M. C. Linder S. (2005). Induction of lysosomal membrane permeabilization by compounds that activate p53-independent apoptosis. Proc. Natl. Acad. Sci. U. S. A. 102, 192–197. 10.1073/pnas.0408592102 15618392 PMC544072

[B31] Esfandiari A. Hawthorne T. A. Nakjang S. Lunec J. (2016). Chemical inhibition of wild-type p53-induced phosphatase 1 (WIP1/ppm1d) by GSK2830371 potentiates the sensitivity to MDM2 inhibitors in a p53-dependent manner. Mol. Cancer Ther. 15, 379–391. 10.1158/1535-7163.MCT-15-0651 26832796 PMC4785723

[B32] Feng Z. Levine A. J. (2010). The regulation of energy metabolism and the IGF-1/mTOR pathways by the p53 protein. Trends Cell Biol. 20, 427–434. 10.1016/j.tcb.2010.03.004 20399660 PMC2921989

[B33] Fitzwalter B. E. Towers C. G. Sullivan K. D. Andrysik Z. Hoh M. Ludwig M. (2018). Autophagy inhibition mediates apoptosis sensitization in cancer therapy by relieving FOXO3a turnover. Dev. Cell 44, 555–565.e3. 10.1016/j.devcel.2018.02.014 29533771 PMC5866042

[B34] Friedler A. Hansson L. O. Veprintsev D. B. Freund S. M. Rippin T. M. Nikolova P. V. (2002). A peptide that binds and stabilizes p53 core domain: chaperone strategy for rescue of oncogenic mutants. Proc. Natl. Acad. Sci. U. S. A. 99, 937–942. 10.1073/pnas.241629998 11782540 PMC117409

[B35] Galluzzi L. Vitale I. Aaronson S. A. Abrams J. M. Adam D. Agostinis P. (2018). Molecular mechanisms of cell death: recommendations of the nomenclature committee on cell death 2018. Cell Death and Differ. 25, 486–541. 10.1038/s41418-017-0012-4 PMC5864239 29362479

[B36] Galmarini C. M. Kamath K. Vanier-Viornery A. Hervieu V. Peiller E. Falette N. (2003). Drug resistance associated with loss of p53 involves extensive alterations in microtubule composition and dynamics. Br. J. Cancer 88, 1793–1799. 10.1038/sj.bjc.6600960 12771997 PMC2377136

[B37] Garg A. D. Krysko D. V. Vandenabeele P. Agostinis P. (2012). The emergence of phox-ER stress induced immunogenic apoptosis. Oncoimmunology 1, 786–788. 10.4161/onci.19750 22934283 PMC3429595

[B38] Ghosh A. Stewart D. Matlashewski G. (2004). Regulation of human p53 activity and cell localization by alternative splicing. Mol. Cell Biol. 24, 7987–7997. 10.1128/MCB.24.18.7987-7997.2004 15340061 PMC515058

[B39] Giannakakou P. Nakano M. Nicolaou K. C. O'Brate A. Yu J. Blagosklonny M. V. (2002). Enhanced microtubule-dependent trafficking and p53 nuclear accumulation by suppression of microtubule dynamics. Proc. Natl. Acad. Sci. U. S. A. 99, 10855–10860. 10.1073/pnas.132275599 12145320 PMC125062

[B40] Giannakakou P. Sackett D. L. Ward Y. Webster K. R. Blagosklonny M. V. Fojo T. (2000). p53 is associated with cellular microtubules and is transported to the nucleus by dynein. Nat. Cell Biol. 2, 709–717. 10.1038/35036335 11025661

[B41] Girardini J. E. Marotta C. Del Sal G. (2014). Disarming mutant p53 oncogenic function. Pharmacol. Res. 79, 75–87. 10.1016/j.phrs.2013.11.003 24246451

[B42] Hanel W. Moll U. M. (2012). Links between mutant p53 and genomic instability. J. Cell Biochem. 113, 433–439. 10.1002/jcb.23400 22006292 PMC4407809

[B43] Hardwick N. R. Frankel P. Ruel C. Kilpatrick J. Tsai W. Kos F. (2018). p53-Reactive T cells are associated with clinical benefit in patients with platinum-resistant epithelial ovarian cancer after treatment with a p53 vaccine and gemcitabine chemotherapy. Clin. Cancer Res. 24, 1315–1325. 10.1158/1078-0432.CCR-17-2709 29301826 PMC5856606

[B44] Hecht J. R. Bedford R. Abbruzzese J. L. Lahoti S. Reid T. R. Soetikno R. M. (2003). A phase I/II trial of intratumoral endoscopic ultrasound injection of ONYX-015 with intravenous gemcitabine in unresectable pancreatic carcinoma. Clin. Cancer Res. 9, 555–561. 12576418

[B45] Hock A. K. Vousden K. H. (2014). The role of ubiquitin modification in the regulation of p53. Biochim. Biophys. Acta 1843, 137–149. 10.1016/j.bbamcr.2013.05.022 23742843

[B46] Hoffman-Luca C. G. Yang C. Y. Lu J. Ziazadeh D. Mceachern D. Debussche L. (2015). Significant differences in the development of acquired resistance to the MDM2 inhibitor SAR405838 between *in vitro* and *in vivo* drug treatment. PLoS One 10, e0128807. 10.1371/journal.pone.0128807 26070072 PMC4466389

[B47] Hu J. Cao J. Topatana W. Juengpanich S. Li S. Zhang B. (2021). Targeting mutant p53 for cancer therapy: direct and indirect strategies. J. Hematol. and Oncol. 14, 157. 10.1186/s13045-021-01169-0 34583722 PMC8480024

[B48] Iulianna T. Kuldeep N. Eric F. (2022). The Achilles' heel of cancer: targeting tumors via lysosome-induced immunogenic cell death. Cell Death Dis. 13, 509. 10.1038/s41419-022-04912-8 35637197 PMC9151667

[B49] Jeffrey P. D. Gorina S. Pavletich N. P. (1995). Crystal structure of the tetramerization domain of the p53 tumor suppressor at 1.7 angstroms. Science 267, 1498–1502. 10.1126/science.7878469 7878469

[B50] Joerger A. C. Ang H. C. Veprintsev D. B. Blair C. M. Fersht A. R. (2005). Structures of p53 cancer mutants and mechanism of rescue by second-site suppressor mutations. J. Biol. Chem. 280, 16030–16037. 10.1074/jbc.M500179200 15703170

[B51] Jonas B. A. Yee K. Koller P. B. Brandwein J. Mims A. S. Michelson G. C. (2022). Preliminary results from a phase 2 open-label, multicenter, dose optimization clinical study of the safety, tolerability, and pharmacokinetic (PK) and pharmacodynamic (PD) profiles of cfi-400945 as a single agent or in combination with azacitidine or decitabine in patients with acute myeloid leukemia, myelodysplastic syndrome or chronic myelomonocytic leukemia (TWT-202). Blood 140, 9076–9077. 10.1182/blood-2022-158370

[B52] Jones R. J. Bjorklund C. C. Baladandayuthapani V. Kuhn D. J. Orlowski R. Z. (2012). Drug resistance to inhibitors of the human double minute-2 E3 ligase is mediated by point mutations of p53, but can be overcome with the p53 targeting agent RITA. Mol. Cancer Ther. 11, 2243–2253. 10.1158/1535-7163.MCT-12-0135 22933706 PMC3469746

[B53] Kadosh E. Snir-Alkalay I. Venkatachalam A. May S. Lasry A. Elyada E. (2020). The gut microbiome switches mutant p53 from tumour-suppressive to oncogenic. Nature 586, 133–138. 10.1038/s41586-020-2541-0 32728212 PMC7116712

[B54] Kandoth C. Mclellan M. D. Vandin F. Ye K. Niu B. Lu C. (2013). Mutational landscape and significance across 12 major cancer types. Nature 502, 333–339. 10.1038/nature12634 24132290 PMC3927368

[B55] Kapralov A. A. Yang Q. Dar H. H. Tyurina Y. Y. Anthonymuthu T. S. Kim R. (2020). Redox lipid reprogramming commands susceptibility of macrophages and microglia to ferroptotic death. Nat. Chem. Biol. 16, 278–290. 10.1038/s41589-019-0462-8 32080625 PMC7233108

[B56] Kaufman H. L. Bommareddy P. K. (2019). Two roads for oncolytic immunotherapy development. J. Immunother. Cancer 7, 26. 10.1186/s40425-019-0515-2 30709365 PMC6359832

[B57] Kim J. H. Yoon E. K. Chung H. J. Park S. Y. Hong K. M. Lee C. H. (2013). p53 acetylation enhances Taxol-induced apoptosis in human cancer cells. Apoptosis 18, 110–120. 10.1007/s10495-012-0772-8 23161364

[B58] Kim E. Giese A. Deppert W. (2009). Wild-type p53 in cancer cells: when a guardian turns into a blackguard. Biochem. Pharmacol. 77, 11–20. 10.1016/j.bcp.2008.08.030 18812169

[B59] Kim J. Yu L. Chen W. Xu Y. Wu M. Todorova D. (2019). Wild-type p53 promotes cancer metabolic switch by inducing PUMA-dependent suppression of oxidative phosphorylation. Cancer Cell 35, 191–203.e8. 10.1016/j.ccell.2018.12.012 30712844

[B60] Kong F. Zou H. Liu X. He J. Zheng Y. Xiong L. (2020). miR-7112-3p targets PERK to regulate the endoplasmic reticulum stress pathway and apoptosis induced by photodynamic therapy in colorectal cancer CX-1 cells. Photodiagnosis Photodyn. Ther. 29, 101663. 10.1016/j.pdpdt.2020.101663 31945549

[B61] Kruse J. P. Gu W. (2009). Modes of p53 regulation. Cell 137, 609–622. 10.1016/j.cell.2009.04.050 19450511 PMC3737742

[B62] Krysko D. V. Garg A. D. Kaczmarek A. Krysko O. Agostinis P. Vandenabeele P. (2012). Immunogenic cell death and DAMPs in cancer therapy. Nat. Rev. Cancer 12, 860–875. 10.1038/nrc3380 23151605

[B63] Ku B. M. Bae Y. H. Koh J. Sun J. M. Lee S. H. Ahn J. S. (2017). Mutational status of TP53 defines the efficacy of Wee1 inhibitor AZD1775 in KRAS-mutant non-small cell lung cancer. Oncotarget 8, 67526–67537. 10.18632/oncotarget.18728 28978051 PMC5620191

[B64] Lacroix M. Riscal R. Arena G. Linares L. K. LE Cam L. (2020). Metabolic functions of the tumor suppressor p53: implications in normal physiology, metabolic disorders, and cancer. Mol. Metab. 33, 2–22. 10.1016/j.molmet.2019.10.002 31685430 PMC7056927

[B65] Lane D. P. (1992). Cancer. p53, guardian of the genome. Nature 358, 15–16. 10.1038/358015a0 1614522

[B66] Lane D. P. Cheok C. F. Lain S. (2010). p53-based cancer therapy. Cold Spring Harb. Perspect. Biol. 2, a001222. 10.1101/cshperspect.a001222 20463003 PMC2926755

[B67] Laptenko O. Prives C. (2006). Transcriptional regulation by p53: one protein, many possibilities. Cell Death Differ. 13, 951–961. 10.1038/sj.cdd.4401916 16575405

[B68] Lau H. C. H. Yu J. (2020). Gut microbiome alters functions of mutant p53 to promote tumorigenesis. Signal Transduct. Target. Ther. 5, 232. 10.1038/s41392-020-00336-y 33037184 PMC7547703

[B69] Lee C. W. Huang C. C. Chi M. C. Lee K. H. Peng K. T. Fang M. L. (2022). Naringenin induces ROS-mediated ER stress, autophagy, and apoptosis in human osteosarcoma cell lines. Molecules 27, 373. 10.3390/molecules27020373 35056691 PMC8781290

[B70] Lee H. B. Ho A. S. Teo S. H. (2006). p53 Status does not affect photodynamic cell killing induced by hypericin. Cancer Chemother. Pharmacol. 58, 91–98. 10.1007/s00280-005-0131-3 16211395

[B71] Lehmann S. Bykov V. J. Ali D. AndréN O. Cherif H. Tidefelt U. (2012). Targeting p53 *in vivo*: a first-in-human study with p53-targeting compound APR-246 in refractory hematologic malignancies and prostate cancer. J. Clin. Oncol. 30, 3633–3639. 10.1200/JCO.2011.40.7783 22965953

[B72] Leijen S. VAN Geel R. M. Sonke G. S. DE Jong D. Rosenberg E. H. Marchetti S. (2016). Phase II study of WEE1 inhibitor AZD1775 plus carboplatin in patients with TP53-mutated ovarian cancer refractory or resistant to first-line therapy within 3 months. J. Clin. Oncol. 34, 4354–4361. 10.1200/JCO.2016.67.5942 27998224

[B73] Leroy B. Anderson M. Soussi T. (2014). TP53 mutations in human cancer: database reassessment and prospects for the next decade. Hum. Mutat. 35, 672–688. 10.1002/humu.22552 24665023

[B74] Leu J. I. Pimkina J. Frank A. Murphy M. E. George D. L. (2009). A small molecule inhibitor of inducible heat shock protein 70. Mol. Cell 36, 15–27. 10.1016/j.molcel.2009.09.023 19818706 PMC2771108

[B75] Levine A. J. (1997). p53, the cellular gatekeeper for growth and division. Cell 88, 323–331. 10.1016/s0092-8674(00)81871-1 9039259

[B76] Levine A. J. (2020a). P53 and the immune response: 40 Years of exploration-A plan for the future. Int. J. Mol. Sci. 21, 541. 10.3390/ijms21020541 31952115 PMC7013403

[B77] Levine A. J. (2020b). p53: 800 million years of evolution and 40 years of discovery. Nat. Rev. Cancer 20, 471–480. 10.1038/s41568-020-0262-1 32404993

[B78] Levine A. J. (2022). Targeting the P53 protein for cancer therapies: the translational impact of P53 research. Cancer Res. 82, 362–364. 10.1158/0008-5472.CAN-21-2709 35110395 PMC8852246

[B79] Liu D. P. Song H. Xu Y. (2010). A common gain of function of p53 cancer mutants in inducing genetic instability. Oncogene 29, 949–956. 10.1038/onc.2009.376 19881536 PMC2837937

[B80] Liu Y. Gu W. (2022a). The complexity of p53-mediated metabolic regulation in tumor suppression. Semin. Cancer Biol. 85, 4–32. 10.1016/j.semcancer.2021.03.010 33785447 PMC8473587

[B81] Liu Y. Gu W. (2022b). p53 in ferroptosis regulation: the new weapon for the old guardian. Cell Death and Differ. 29, 895–910. 10.1038/s41418-022-00943-y PMC9091200 35087226

[B82] Li Y. Zou L. Li Q. Haibe-Kains B. Tian R. Li Y. (2010). Amplification of LAPTM4B and YWHAZ contributes to chemotherapy resistance and recurrence of breast cancer. Nat. Med. 16, 214–218. 10.1038/nm.2090 20098429 PMC2826790

[B83] Lozano G. (2019). Restoring p53 in cancer: the promises and the challenges. J. Mol. Cell Biol. 11, 615–619. 10.1093/jmcb/mjz063 31283825 PMC6736346

[B84] Ma Y. M. Peng Y. M. Zhu Q. H. Gao A. H. Chao B. He Q. J. (2016). Novel CHOP activator LGH00168 induces necroptosis in A549 human lung cancer cells via ROS-mediated ER stress and NF-κB inhibition. Acta Pharmacol. Sin. 37, 1381–1390. 10.1038/aps.2016.61 27264312 PMC5057234

[B85] Mabjeesh N. J. Escuin D. Lavallee T. M. Pribluda V. S. Swartz G. M. Johnson M. S. (2003). 2ME2 inhibits tumor growth and angiogenesis by disrupting microtubules and dysregulating HIF. Cancer Cell 3, 363–375. 10.1016/s1535-6108(03)00077-1 12726862

[B86] Malley K. R. Koroleva O. Miller I. Sanishvili R. Jenkins C. M. Gross R. W. (2018). The structure of iPLA_2_β reveals dimeric active sites and suggests mechanisms of regulation and localization. Nat. Commun. 9, 765. 10.1038/s41467-018-03193-0 29472584 PMC5823874

[B87] Mandinova A. Lee S. W. (2011). The p53 pathway as a target in cancer therapeutics: obstacles and promise. Sci. Transl. Med. 3, 64rv1. 10.1126/scitranslmed.3001366 PMC3763710 21209413

[B88] Mantovani F. Collavin L. Del Sal G. (2019). Mutant p53 as a guardian of the cancer cell. Cell Death Differ. 26, 199–212. 10.1038/s41418-018-0246-9 30538286 PMC6329812

[B89] Milner J. (1995). Flexibility: the key to p53 function? Trends Biochem. Sci. 20, 49–51. 10.1016/s0968-0004(00)88954-9 7701559

[B90] Montesinos P. Beckermann B. M. Catalani O. Esteve J. Gamel K. Konopleva M. Y. (2020). MIRROS: a randomized, placebo-controlled, Phase III trial of cytarabine ± idasanutlin in relapsed or refractory acute myeloid leukemia. Future Oncol. 16, 807–815. 10.2217/fon-2020-0044 32167393

[B91] Mrakovcic M. FröHLICH L. F. (2018). p53-Mediated molecular control of autophagy in tumor cells. Biomolecules 8, 14. 10.3390/biom8020014 29561758 PMC6022997

[B92] New M. Olzscha H. LA Thangue N. B. (2012). HDAC inhibitor-based therapies: can we interpret the code? Mol. Oncol. 6, 637–656. 10.1016/j.molonc.2012.09.003 23141799 PMC5528347

[B93] Nguyen H. A. Vu S. H. Jung S. Lee B. S. Nguyen T. N. Q. Lee H. (2022). SERTAD1 sensitizes breast cancer cells to doxorubicin and promotes lysosomal protein biosynthesis. Biomedicines 10, 1148. 10.3390/biomedicines10051148 35625886 PMC9139069

[B94] Nikolova P. V. Wong K. B. Dedecker B. Henckel J. Fersht A. R. (2000). Mechanism of rescue of common p53 cancer mutations by second-site suppressor mutations. Embo J. 19, 370–378. 10.1093/emboj/19.3.370 10654936 PMC305574

[B95] Nitzsche B. HöPFNER M. Biersack B. (2023). Synthetic small molecule modulators of Hsp70 and Hsp40 chaperones as promising anticancer agents. Int. J. Mol. Sci. 24, 4083. 10.3390/ijms24044083 36835501 PMC9964478

[B96] Olivier M. Eeles R. Hollstein M. Khan M. A. Harris C. C. Hainaut P. (2002). The IARC TP53 database: new online mutation analysis and recommendations to users. Hum. Mutat. 19, 607–614. 10.1002/humu.10081 12007217

[B97] Opyrchal M. Aderca I. Galanis E. (2009). Phase I clinical trial of locoregional administration of the oncolytic adenovirus ONYX-015 in combination with mitomycin-C, doxorubicin, and cisplatin chemotherapy in patients with advanced sarcomas. Methods Mol. Biol. 542, 705–717. 10.1007/978-1-59745-561-9_35 19565928 PMC3180922

[B98] Park S. H. Baek K. H. Shin I. Shin I. (2018). Subcellular Hsp70 inhibitors promote cancer cell death via different mechanisms. Cell Chem. Biol. 25, 1242–1254.e8. 10.1016/j.chembiol.2018.06.010 30057298

[B99] Peng X. Zhang M. Q. Conserva F. Hosny G. Selivanova G. Bykov V. J. (2013). APR-246/PRIMA-1MET inhibits thioredoxin reductase 1 and converts the enzyme to a dedicated NADPH oxidase. Cell Death Dis. 4, e881. 10.1038/cddis.2013.417 24157875 PMC3920950

[B100] Pishas K. I. Adwal A. Neuhaus S. J. Clayer M. T. Farshid G. Staudacher A. H. (2015). XI-006 induces potent p53-independent apoptosis in Ewing sarcoma. Sci. Rep. 5, 11465. 10.1038/srep11465 26095524 PMC4476092

[B101] Polyak K. Xia Y. Zweier J. L. Kinzler K. W. Vogelstein B. (1997). A model for p53-induced apoptosis. Nature 389, 300–305. 10.1038/38525 9305847

[B102] Raycroft L. Wu H. Y. Lozano G. (1990). Transcriptional activation by wild-type but not transforming mutants of the p53 anti-oncogene. Science 249, 1049–1051. 10.1126/science.2144364 2144364 PMC2935288

[B103] Sallman D. A. Dezern A. E. Steensma D. P. Sweet K. L. Cluzeau T. Sekeres M. A. (2018). Phase 1b/2 combination study of APR-246 and azacitidine (AZA) in patients with TP53 mutant myelodysplastic syndromes (MDS) and acute myeloid leukemia (AML). Blood 132, 3091. 10.1182/blood-2018-99-119990

[B104] Scherz-Shouval R. Weidberg H. Gonen C. Wilder S. Elazar Z. Oren M. (2010). p53-dependent regulation of autophagy protein LC3 supports cancer cell survival under prolonged starvation. Proc. Natl. Acad. Sci. U. S. A. 107, 18511–18516. 10.1073/pnas.1006124107 20937856 PMC2972967

[B105] Seervi M. Rani A. Sharma A. K. Santhosh Kumar T. R. (2018). ROS mediated ER stress induces Bax-Bak dependent and independent apoptosis in response to Thioridazine. Biomed. Pharmacother. 106, 200–209. 10.1016/j.biopha.2018.06.123 29960166

[B106] Shim D. Duan L. Maki C. G. (2021). P53-regulated autophagy and its impact on drug resistance and cell fate. Cancer Drug Resist 4, 85–95. 10.20517/cdr.2020.85 34532654 PMC8443158

[B107] Shu H. K. Kim M. M. Chen P. Furman F. Julin C. M. Israel M. A. (1998). The intrinsic radioresistance of glioblastoma-derived cell lines is associated with a failure of p53 to induce p21(BAX) expression. Proc. Natl. Acad. Sci. U. S. A. 95, 14453–14458. 10.1073/pnas.95.24.14453 9826721 PMC24394

[B108] Soliman H. Khambati F. Han H. S. Ismail-Khan R. Bui M. M. Sullivan D. M. (2018). A phase-1/2 study of adenovirus-p53 transduced dendritic cell vaccine in combination with indoximod in metastatic solid tumors and invasive breast cancer. Oncotarget 9, 10110–10117. 10.18632/oncotarget.24118 29515795 PMC5839376

[B109] Song H. Hollstein M. Xu Y. (2007). p53 gain-of-function cancer mutants induce genetic instability by inactivating ATM. Nat. Cell Biol. 9, 573–580. 10.1038/ncb1571 17417627

[B110] Soula M. Weber R. A. Zilka O. Alwaseem H. LA K. Yen F. (2020). Metabolic determinants of cancer cell sensitivity to canonical ferroptosis inducers. Nat. Chem. Biol. 16, 1351–1360. 10.1038/s41589-020-0613-y 32778843 PMC8299533

[B111] Suh Y.-A. Post S. M. Elizondo-Fraire A. C. Maccio D. R. Jackson J. G. EL-Naggar A. K. (2011). Multiple stress signals activate mutant p53 *in vivo* . Cancer Res. 71, 7168–7175. 10.1158/0008-5472.CAN-11-0459 21983037 PMC3320147

[B112] Sun Y. Xia P. Zhang H. Liu B. Shi Y. (2016). P53 is required for Doxorubicin-induced apoptosis via the TGF-beta signaling pathway in osteosarcoma-derived cells. Am. J. Cancer Res. 6, 114–125. 27073729 PMC4759403

[B113] Synnott N. C. O'Connell D. Crown J. Duffy M. J. (2020). COTI-2 reactivates mutant p53 and inhibits growth of triple-negative breast cancer cells. Breast Cancer Res. Treat. 179, 47–56. 10.1007/s10549-019-05435-1 31538264

[B114] Taritsa I. Fossel E. Neote K. (2022). Abstract 4244: using lysosomal immunogenic cell death to target cancer via xanthine oxidase. Cancer Res. 82, 4244. 10.1158/1538-7445.am2022-4244

[B115] Taura M. Kariya R. Kudo E. Goto H. Iwawaki T. Amano M. (2013). Comparative analysis of ER stress response into HIV protease inhibitors: lopinavir but not darunavir induces potent ER stress response via ROS/JNK pathway. Free Radic. Biol. Med. 65, 778–788. 10.1016/j.freeradbiomed.2013.08.161 23973637

[B116] Terenzi A. Pirker C. Keppler B. K. Berger W. (2016). Anticancer metal drugs and immunogenic cell death. J. Inorg. Biochem. 165, 71–79. 10.1016/j.jinorgbio.2016.06.021 27350082

[B117] Tewari K. S. Eskander R. N. Monk B. J. (2015). Development of olaparib for BRCA-deficient recurrent epithelial ovarian cancer. Clin. Cancer Res. 21, 3829–3835. 10.1158/1078-0432.CCR-15-0088 26169965

[B118] Tolcher A. Mamdani H. Chalasani P. Meric-Bernstam F. Gazdoiu M. Makris L. (2021). Abstract CT016: clinical activity of single-agent ZN-c3, an oral WEE1 inhibitor, in a phase 1 dose-escalation trial in patients with advanced solid tumors. Cancer Res. 81, CT016. 10.1158/1538-7445.am2021-ct016

[B119] Tomicic M. T. Dawood M. Efferth T. (2021). Epigenetic alterations upstream and downstream of p53 signaling in colorectal carcinoma. Cancers (Basel) 13, 4072. 10.3390/cancers13164072 34439227 PMC8394868

[B120] Topatana W. Juengpanich S. Li S. Cao J. Hu J. Lee J. (2020). Advances in synthetic lethality for cancer therapy: cellular mechanism and clinical translation. J. Hematol. Oncol. 13, 118. 10.1186/s13045-020-00956-5 32883316 PMC7470446

[B121] Turubanova V. D. Balalaeva I. V. Mishchenko T. A. Catanzaro E. Alzeibak R. Peskova N. N. (2019). Immunogenic cell death induced by a new photodynamic therapy based on photosens and photodithazine. J. Immunother. Cancer 7, 350. 10.1186/s40425-019-0826-3 31842994 PMC6916435

[B122] Vasey P. A. Shulman L. N. Campos S. Davis J. Gore M. Johnston S. (2002). Phase I trial of intraperitoneal injection of the E1B-55-kd-gene-deleted adenovirus ONYX-015 (dl1520) given on days 1 through 5 every 3 weeks in patients with recurrent/refractory epithelial ovarian cancer. J. Clin. Oncol. 20, 1562–1569. 10.1200/JCO.2002.20.6.1562 11896105

[B123] Veitch Z. W. Cescon D. W. Denny T. Yonemoto L. M. Fletcher G. Brokx R. (2019). Safety and tolerability of CFI-400945, a first-in-class, selective PLK4 inhibitor in advanced solid tumours: a phase 1 dose-escalation trial. Br. J. Cancer 121, 318–324. 10.1038/s41416-019-0517-3 31303643 PMC6738068

[B124] Venkatesh D. O'Brien N. A. Zandkarimi F. Tong D. R. Stokes M. E. Dunn D. E. (2020). MDM2 and MDMX promote ferroptosis by PPARα-mediated lipid remodeling. Genes Dev. 34, 526–543. 10.1101/gad.334219.119 32079652 PMC7111265

[B125] Ventura A. Kirsch D. G. Mclaughlin M. E. Tuveson D. A. Grimm J. Lintault L. (2007). Restoration of p53 function leads to tumour regression *in vivo* . Nature 445, 661–665. 10.1038/nature05541 17251932

[B126] Veprintsev D. B. Freund S. M. Andreeva A. Rutledge S. E. Tidow H. CañADILLAS J. M. (2006). Core domain interactions in full-length p53 in solution. Proc. Natl. Acad. Sci. U. S. A. 103, 2115–2119. 10.1073/pnas.0511130103 16461914 PMC1413758

[B127] Vinyals A. Peinado M. A. Gonzalez-Garrigues M. Monzó M. Bonfil R. D. Fabra A. (1999). Failure of wild-type p53 gene therapy in human cancer cells expressing a mutant p53 protein. Gene Ther. 6, 22–33. 10.1038/sj.gt.3300786 10341872

[B128] Wade M. Wang Y. V. Wahl G. M. (2010). The p53 orchestra: mdm2 and Mdmx set the tone. Trends Cell Biol. 20, 299–309. 10.1016/j.tcb.2010.01.009 20172729 PMC2910097

[B129] Wang Z.-X. Ma J. Li X.-Y. Wu Y. Shi H. Chen Y. (2021). Quercetin induces p53-independent cancer cell death through lysosome activation by the transcription factor EB and Reactive Oxygen Species-dependent ferroptosis. Br. J. Pharmacol. 178, 1133–1148. 10.1111/bph.15350 33347603

[B130] Wang Q. Ju X. Wang J. Fan Y. Ren M. Zhang H. (2018). Immunogenic cell death in anticancer chemotherapy and its impact on clinical studies. Cancer Lett. 438, 17–23. 10.1016/j.canlet.2018.08.028 30217563

[B131] Wang S. Konorev E. A. Kotamraju S. Joseph J. Kalivendi S. Kalyanaraman B. (2004). Doxorubicin induces apoptosis in normal and tumor cells via distinctly different mechanisms. intermediacy of H(2)O(2)- and p53-dependent pathways. J. Biol. Chem. 279, 25535–25543. 10.1074/jbc.M400944200 15054096

[B132] Wang X. Guo Q. Tao L. Zhao L. Chen Y. An T. (2017). E platinum, a newly synthesized platinum compound, induces apoptosis through ROS-triggered ER stress in gastric carcinoma cells. Mol. Carcinog. 56, 218–231. 10.1002/mc.22486 27061377

[B133] Welch J. S. Petti A. A. Miller C. A. Fronick C. C. O'Laughlin M. Fulton R. S. (2016). TP53 and decitabine in acute myeloid leukemia and myelodysplastic syndromes. N. Engl. J. Med. 375, 2023–2036. 10.1056/NEJMoa1605949 27959731 PMC5217532

[B134] Wischhusen J. Naumann U. Ohgaki H. Rastinejad F. Weller M. (2003). CP-31398, a novel p53-stabilizing agent, induces p53-dependent and p53-independent glioma cell death. Oncogene 22, 8233–8245. 10.1038/sj.onc.1207198 14614447

[B135] Wong K. B. Dedecker B. S. Freund S. M. Proctor M. R. Bycroft M. Fersht A. R. (1999). Hot-spot mutants of p53 core domain evince characteristic local structural changes. Proc. Natl. Acad. Sci. U. S. A. 96, 8438–8442. 10.1073/pnas.96.15.8438 10411893 PMC17534

[B136] Wong S. H. Yu J. (2019). Gut microbiota in colorectal cancer: mechanisms of action and clinical applications. Nat. Rev. Gastroenterology and Hepatology 16, 690–704. 10.1038/s41575-019-0209-8 31554963

[B137] Xie D. Wang Q. Wu G. (2022). Research progress in inducing immunogenic cell death of tumor cells. Front. Immunol. 13, 1017400. 10.3389/fimmu.2022.1017400 36466838 PMC9712455

[B138] Yuan X. M. Li W. Dalen H. Lotem J. Kama R. Sachs L. (2002). Lysosomal destabilization in p53-induced apoptosis. Proc. Natl. Acad. Sci. U. S. A. 99, 6286–6291. 10.1073/pnas.092135599 11959917 PMC122941

[B139] Zeimet A. G. Marth C. (2003). Why did p53 gene therapy fail in ovarian cancer? Lancet Oncol. 4, 415–422. 10.1016/s1470-2045(03)01139-2 12850192

[B140] Zhang W. W. Li L. Li D. Liu J. Li X. Li W. (2018). The first approved gene therapy product for cancer ad-p53 (gendicine): 12 Years in the clinic. Hum. Gene Ther. 29, 160–179. 10.1089/hum.2017.218 29338444

[B141] Zhang Z. J. Wang K. P. Mo J. G. Xiong L. Wen Y. (2020). Photodynamic therapy regulates fate of cancer stem cells through reactive oxygen species. World J. Stem Cells 12, 562–584. 10.4252/wjsc.v12.i7.562 32843914 PMC7415247

[B142] Zhang B. Tang C. Yao Y. Chen X. Zhou C. Wei Z. (2021). The tumor therapy landscape of synthetic lethality. Nat. Commun. 12, 1275. 10.1038/s41467-021-21544-2 33627666 PMC7904840

[B143] Zhu H. Gao H. Ji Y. Zhou Q. DU Z. Tian L. (2022). Targeting p53-MDM2 interaction by small-molecule inhibitors: learning from MDM2 inhibitors in clinical trials. J. Hematol. Oncol. 15, 91. 10.1186/s13045-022-01314-3 35831864 PMC9277894

